# Preserved Calretinin Interneurons in an *App* Model of Alzheimer’s Disease Disrupt Hippocampal Inhibition via Upregulated P2Y1 Purinoreceptors

**DOI:** 10.1093/cercor/bhz165

**Published:** 2019-08-13

**Authors:** Anqi Shi, Alexandra L Petrache, Jiachen Shi, Afia B Ali

**Affiliations:** UCL School of Pharmacy, London, WC1N 1AX, UK

**Keywords:** Alzheimer’s disease, astrocytes, interneurons, P2Y1 receptors, synapse

## Abstract

To understand the pathogenesis of specific neuronal circuit dysfunction in Alzheimer’s disease (AD), we investigated the fate of three subclasses of “modulatory interneurons” in hippocampal CA1 using the *App^NL-F/NL-F^* knock-in mouse model of AD. Cholecystokinin- and somatostatin-expressing interneurons were aberrantly hyperactive preceding the presence of the typical AD hallmarks: neuroinflammation and amyloid-β (Aβ) accumulation. These interneurons showed an age-dependent vulnerability to Aβ penetration and a reduction in density and coexpression of the inhibitory neurotransmitter GABA synthesis enzyme, glutamic acid decarboxylase 67 (GAD67), suggesting a loss in their inhibitory function. However, calretinin (CR) interneurons—specialized to govern *only* inhibition, showed resilience to Aβ accumulation, preservation of structure, and displayed synaptic hyperinhibition, despite the lack of inhibitory control of CA1 excitatory pyramidal cells from midstages of the disease. This aberrant inhibitory homeostasis observed in CA1 CR cells and pyramidal cells was “normalized” by blocking P2Y1 purinoreceptors, which were “upregulated” and strongly expressed in CR cells and astrocytes in *App^NL-F/NL-F^* mice in the later stages of AD. In summary, AD-associated cell-type selective destruction of inhibitory interneurons and disrupted inhibitory homeostasis rectified by modulation of the upregulated purinoreceptor system may serve as a novel therapeutic strategy to normalize selective dysfunctional synaptic homeostasis during pathogenesis of AD.

## Introduction

Idiopathic and familial Alzheimer’s disease (AD) are debilitating chronic neurodegenerative conditions characterized by accumulation of amyloid-β (Aβ) plaques, neurofibrillary tangles, dystrophic neurites, and gliosis (Holtzman et al. 2011), as well as progressive cognitive deficits leading to neurodegeneration and abnormal aging.

The Cornu Ammonis (CA) 1 region of the hippocampus together with the neighboring cortical region, the entorhinal cortex, is one of the significant brain regions that play a critical role in memory formation and retrieval and one of the initial regions to be disrupted in early AD. This region is enriched with pathways that are heavily innervated by the diverse inhibitory gamma-aminobutyric acid (GABA)–containing interneuron populations. The GABA_A_ receptor family is known to play a vital role in cognitive functions, including learning and memory. Although there are currently 22 known subgroups of interneurons that perform distinct functions through activation of postsynaptic GABA_A_ receptors (Klausberger and Somogyi 2008), the identity of the vulnerable and resistant subclasses of interneurons in AD needs detailed investigation.

This knowledge gap will help us understand a consistent observation that spans from human studies to various rodent models of AD, that is, the abnormal synaptic hyperexcitation preceding phenotypic alteration of the disease, which has been noted as a relevant therapeutic target (Palop et al. 2007; Busche et al. 2008; Khan et al. 2014). It has been suggested that AD-related cortical neurodegeneration is associated with overexcitation of hippocampal activity (Putcha et al. 2011), which is consistent with various in vitro and in vivo models of AD, demonstrating that increased neuronal activity stimulates tau release which further enhances tau pathology (Wu et al. 2016; Yamada et al. 2014), as well as augmentation of Aβ depositions from presynaptic terminals (Yamamoto et al. 2015). Using the first *App* knock-in mouse model of AD, *App^NL-F/NL-F^*, we recently reported a time-dependent spread of synaptic hyperexcitability initiated in the entorhinal cortex that spreads to other cortical regions, altering the balance of excitation–inhibition in the AD model (Petrache et al. 2019). This synaptic hyperexcitation presents a paradox in AD, as others have reported that inhibitory postsynaptic GABA_A_ receptors that govern excitation in memory-related pathways remain preserved in human brains of AD patients (Howell et al. 2000; Rissman et al. 2007).

Furthermore, recently, it has become apparent that the microcircuit pathogenesis of AD is more complex than just synaptic loss, but associated with a combination of an abnormal hyperactive interplay between neurons, and their synaptic support system—astrocytes and glial cells (Heneka et al. 2015; Palop and Mucke 2016b)—which mediate their activity through P2Y1 purinoreceptor (P2Y1R) pathways (Reichenbach et al. 2018). However, the pathophysiological effects of Aβ on the interactions of specific microcircuits with the neuronal support system remains to be fully elucidated in the field, and filling this missing gap will fulfill an unmet demand in the dementia field leading to an advance in our understanding of the underlying pathogenesis of the disease to bring us a step closer to designing early-stage therapeutic intervention to prevent or halt the disease progression.

In the present study, we asked the question of whether there is a cell-type selective destruction of inhibitory interneurons responsible for fine-tuning local circuitry in AD and whether specific neuronal populations were vulnerable to Aβ association or another key detrimental factor associated with the pathogenies of AD. Using the *App^NL-F/NL-F^* mouse model of AD and neuroanatomy combined with electrophysiology, we focused on investigating three subtypes of dendrite-targeting modulatory interneurons in CA1, namely, cholecystokinin (CCK)-expressing, somatostatin (SST)-expressing, and the previously “unexplored”, disinhibitory calretinin (CR) circuitry. The CR—containing interneurons are a major part of the disinhibitory network governing other inhibitory cells (Gulyas et al. 1996). We hypothesize that an abnormal CR microcircuitry is the key candidate mechanism for the paradoxical hyperexcitability associated with AD and that correction of this abnormal circuit behavior by blocking overactive P2Y1Rs could offer a novel therapeutic strategy for preventing, ultimately, neurodegeneration in AD.

## Methods

### Animals

#### Experimental Animals

All of the procedures in this study were carried out in accordance with the British Home Office regulations under the Animal Scientific Procedure Act 1986, under the project license PPL: P1ADA633A held by the principal investigator, Dr Afia Ali. All procedures were approved by both internal and external UCL ethics committees and in accordance with the ARRIVE guidelines for reporting experiments involving animals (McGrath et al. 2010). A total of ~ 85 animals (disease model and wild-type) were used in this study. The animals had ad libitum access to food and water and were reared in cages of maximum five inhabitants, with a day:night cycle of 12 h:12 h.

The knock-in *APP^NL-F/NL-F^* AD mouse model was used for experiments (Saito et al. 2014). This particular mouse model was chosen because it follows the progression of human AD more faithfully. Since amyloid β-peptide (Aβ) plaque deposition is a key AD pathological hallmark, the model exhibits pathogenic Aβ accumulation while also maintaining biological amyloid precursor protein (APP) levels without overexpression artifacts. The *APP^NL-F^* model consists of the introduction of two familial AD (FAD) mutations: KM670/671NL and I716F. The former, identified as the Swedish mutation, increases β-site cleavage of APP to produce elevated amounts of both Aβ_40_ and Aβ_42_, whereas the latter, known as the Beyreuther/Iberian mutation, promotes γ-site cleavage at C-terminal position 42, thereby increasing the Aβ_42_/Aβ_40_ ratio in favor of the more hydrophobic Aβ_42_ (Saito et al. 2014). Both features are key to the integrity of the disease phenotype. The knock-in line was crossed with C57BL/6 mice, and the resulting heterozygous pairs were used for breeding, but excluded from experiments. Only male *APP^NL-F/NL-F^* and age-matched wild-type (C57BL/6) mice from the same breeding were used as control. *APP^NL-F/NL-F^* and control mice were investigated at three different ages, grouped into three age groups where no differences was observed within the time window; these were 1–3 months, 4–6 months, and 9–18 months.

Animals were genotyped via standard polymerase chain reaction using the following four primers: 5′-ATCTCGGAAGTGAAGATG-3′, 5′-TGTAGATGAGAACTTAAC-3′, 5′-ATCTCGGAAGTGAATCTA-3′, and 5′-CGTATAATGTATGCTATACGAAG-3′ as previously described (Saito et al. 2014).

### Tissue Collection and Preparation

Mice were anesthetized by an intraperitoneal injection of 60 mg/kg phenobarbitone and perfused transcardially with artificial cerebrospinal fluid (ACSF) containing sucrose. The level of anesthesia was monitored using pedal and tail pinch reflexes, rate, depth, and pattern of respiration through observation and color of mucous membranes and skin. The ACSF comprised (in mM) 248 sucrose, 3.3 KCl, 1.4 NaH_2_PO_4_, 2.5 CaCl_2_, 1.2 MgCl_2_, 25.5 NaHCO_3_, and 15 glucose, which was bubbled with 95% O_2_ and 5% CO_2_. The animals were then decapitated and the brain removed, and coronal slices of the cortex and hippocampus—300 μm thick—were cut in ice-cold standard ACSF using an automated vibratome (Leica, Germany). This standard ACSF contained (in mM) 121 NaCl, 2.5 KCl, 1.3 NaH_2_PO_4_, 2 CaCl_2_, 1 MgCl_2_, 20 glucose, and 26 NaHCO_3_, equilibrated with 95% O_2_ and 5% CO_2_. Slices were incubated in ACSF for 1 h at room temperature (20–23 °C) prior to recording. Brain slices were placed in a submerged chamber and superperfused with ACSF at a rate of 1–2 mL min^−1^ for electrophysiological recordings. For neuroanatomical studies, brains were immediately fixed after perfusion in 4% paraformaldehyde plus 0.2% picric acid in 0.1 M phosphate buffer (PB) for 24 h prior to sectioning.

### Electrophysiology

Whole-cell somatic recordings were performed using patch electrodes with resistances of 8–11 MΩ made from filamented borosilicate glass capillaries (Harvard Apparatus, United Kingdom) and filled with a solution containing (in mM) 134 K gluconate, 10 HEPES, 10 phosphocreatine, 2 Na_2_ATP, 0.2 Na_2_GTP, and 0.2% w/v biocytin.

CA1 pyramidal cells and interneurons in the stratum oriens, stratum radiatum, and stratum lacunosum moleculare were selected for recording based on the shape of their soma using video microscopy under near infrared differential interference contrast illumination. Cells were further characterized by their electrophysiological properties obtained from injecting a series of 500-ms depolarizing and hyperpolarizing current pulses. Action potential parameters were measured from responses to depolarizing current steps (+25–150 pA, 500 ms), which induced a single or a train of action potentials. The input resistance and membrane time constant were determined from voltage changes in response to hyperpolarizing current steps (−100 pA, 500 ms). Recorded cells were filled with biocytin dye, and neurons were further identified based on their gross morphology (see below).

Spontaneous postsynaptic potentials were recorded from passive membrane responses, and mixed spontaneous excitatory postsynaptic potentials (sEPSPs) and spontaneous inhibitory postsynaptic potentials (sIPSPs) were collected in 60-s frame samples, repeated at 0.33 Hz. Recordings were carried out under the current clamp mode of operation (NPI SEC-05LX amplifier; NPI Electronic, Germany), low pass filtered at 2 kHz, and digitized at 5 kHz using a CED 1401 interface (Cambridge Electronic Design, United Kingdom). Input resistance was monitored throughout experiments by means of a hyperpolarizing current step (−0.001 nA, 10 ms). Signal (Cambridge Electronic Design, United Kingdom) was used to acquire recordings and generate current steps. The average amplitudes of spontaneous events and their frequency were measured manually from single sweep data sets of 60-s recordings, including a total sweep range of 30–50 frames (i.e., 30–50 min of recording); synaptic noise was taken as ±0.15 mV from baseline; for example, values above +0.15 mV were considered as synaptic events.

For in vitro pharmacological studies, P2Y1 receptor modulators—MRS2365 (agonist, 0.5–1 μM, Tocris Bioscience, United Kingdom) and BPTU (inhibitor, 0.5–1 μM, Tocris Bioscience, United Kingdom)—were bath-applied. Average data points after drug application were obtained after steady-state responses were attained with the drugs, which was ~ 15–20 min after onset of the bath application.

### Neuroanatomical Procedures and Analysis

#### Recovery of Biocytin-Labeled Cells Post Electrophysiological Recordings

After electrophysiological recordings with pharmacological protocols, the slices were only suitable for biocytin recovery due to the long recording in the range of 45–90 min. Slices were fixed in 4% paraformaldehyde plus 0.2% picric acid in 0.1 M phosphate buffer (PB) for 24 h and then resectioned at 70 μm. Slices were then incubated in avidin–biotin complex (ABC) overnight at 4 °C, followed by the DAB protocol. Anatomically recovered cells were reconstructed manually from consecutive slices at ×100 objective under a Leica DMR microscope with an attached drawing tube.

#### Immunofluorescence Procedures, Confocal Image Acquisition, and Analysis

Coronal sections containing the neocortex and hippocampal formation were sectioned at 100-μm thickness using a vibratome (Vibroslice, Campden Instruments, Loughborough, United Kingdom) and placed in a 24-well plate containing 10% phosphate buffer (PB). Each experiment consisted of slices from wild-type and *APP^NL-F/NL-F^* age-matched mice and kept in separate 24-well plates. The sections per brain were allocated to the antibody and procedure, but all sections underwent identical protocols for either immunofluorescence or immunoperoxidase procedures. Prior to these specific procedures, all sections were washed in 0.3% Triton X-100 in Tris-buffered saline (TBS-T), followed by incubation in 1% hydrogen peroxide aqueous solution for 30 min. After further rinses in TBS-T, sections were incubated in phosphate-buffered saline (PBS) containing 10% normal goat serum (Sigma-Aldrich, United States of America) for 1 h at room temperature. This followed incubation in the specific primary antibodies to target the desired proteins shown in Table 1.

**Table 1:** Antibody and serum information

Primary antibodies			
Antibody target	Company	Species	Dilution	Serum
Cholecystokinin	Frontier Institute	Rabbit	1:750	NHS
Calretinin	Swant	Goat	1:1000	NDS
Somatostatin	Santa Cruz Biotechnology	Rabbit	1:200	NHS
GAD67	Millipore	Mouse	1:2000	NHS
APP695	Thermo Fisher	Mouse	1:1000 for both IF and IP	NHS
CD68	Bio-Rad	Goat	1:500; 1:3000 for IP	NGS
GFAP	Agilent (Dako)	Rabbit	1:500; 1:2000 for IP	NGS
Secondary antibodies				
Immunofluorescence				
Texas Red	Thermo Scientific	Rabbit	1:500	
FITC	Sigma-Aldrich	Mouse	1:875	
Alexa 488	Abcam	Rabbit	1:1000	
Alexa 568	Molecular Probes	Goat	1:500	
DAPI	Sigma-Aldrich	Multiple	1:1000	
Immunoperoxidase				
Biotinylated	Vector Laboratories	Mouse, goat, rabbit	1:500	

IF, immunofluorescence; IP, immunoperoxidase; NGS, normal goat serum.

The “Serum” column refers to the serum used for the 1-h incubation step before placement in primary antibodies and the serum used in conjunction with the secondary antibody solution.

When colocalization assessments were performed, both primary antibodies were added to the same well simultaneously.

Slices were incubated in primary antibody for 48 h on a platform shaker at 4 °C. Afterwards, the sections were washed (0.3% TBS-T, 3 × 10 min) and incubated in the appropriate secondary antibodies (see Table 1) for 3 h. The secondary antibody solution also contained the appropriate serum in rough proportion of 0.05% of the total solution volume. When two fluorophores were added to the same well, the serum used was normal horse serum (NHS), with the exception of solutions applied to wells stained for CR, for which normal donkey serum (NDS) was used. The sections were then washed (0.3% TBS-T, 3 × 10 min), and slices stained with CD68 and glial fibrillary acidic protein (GFAP) were incubated with DAPI (1:1000) for 15 min. After further washes, the slices were mounted on glass slides using the antifade mounting medium Vectashield (Vector Lab, United Kingdom).

Images were acquired with an LSM 710 confocal microscope and processed using Zeiss ZEN Black 2009 software. *Z*-stacks of the CA1 region were taken at ×20 magnification. The first and last 10 μm were discarded from each section to prevent repeated capture of the same cell. When needed, more than one image was taken per slice so as to obtain an accurate average measurement for the region.

To distinguish cells from any background fluorescence, a threshold was calculated by summing the mean intensity of the collapsed *Z*-stack and twice the standard deviation, using ZEN 2009. Colocalization was confirmed when fluorophores marking both the cell of interest and GAD67 or P2Y1 receptor were at an intensity that exceeded the threshold and when GAD67 was present within the cell outline. Somata that exceeded the threshold were counted, and the total number obtained was then divided by the volume of the *Z*-stack (4.99 × 10^−2^ mm^3^) to determine the density of cell fluorescence + GAD67-coexpressing interneurons. GAD67 or P2Y1 receptor colocalization with CR cells was calculated similarly, by analyzing the colocalization coefficient obtained using ZEN 2009.

P2Y1R expression colocalized with either CR, GFAP, or CaMKII-α was estimated using the correlation coefficient *R*, which was calculated with ZEN 2009. The closer *R* is to 1, the stronger the positive correlation between the two variables. *Z*-stacks were collapsed into one image and a region-of-interest (ROI)–based analysis was used to quantify receptor colocalization with the cell type of interest. In the “Coloc” tab, the colocalization crosshairs were set using a threshold calculated with values obtained from “Histo” using the formula Threshold = mean + (standard deviation / 2). Then, the channel for the structure of interest was turned on and the cell of interest was outlined. Afterwards, the P2Y1R channel was turned on and *R* was calculated by the software.

#### Immunoperoxidase Procedure and Analysis

After washes in TBS-T, the sections were incubated in secondary biotinylated antibodies (see Table 1). After incubation with the secondary biotinylated antibody and after washes in TBS-T, there was a further incubation in avidin–biotin complex (ABC)–horseradish peroxidase (Vector Laboratories, United Kingdom) solution, for 2 h at room temperature. The sections were then washed further in TBS-T and processed with 3,3′-diaminobenzidine (DAB) and subsequently dehydrated and mounted (Khan et al. 2018).

The darkness density of slices was measured using the Fiji imaging package. DAB-stained pictures were taken under ×10 light microscope, and the background was kept consistent. Pictures were processed by color deconvolution and “H DAB,” and the “mean gray value” was used to measure the darkness density. Mean gray values were normalized into optical density numbers by the formula OD = log (max intensity / mean intensity), where max intensity = 255 for 8-bit images.

### Statistics

All figures displaying error bars represent the standard deviation from the mean. The “n” is given as the number of observations and the number of animals used, unless otherwise stated.

Various statistical tests were performed depending on the parameter analyzed; each figure legends detail the specific statistical test used. For example, two-sampled unpaired Student’s *t*-test was used to compare biophysical parameters between wild-type and *App^NL-F/NL-F^* mice. A two-way ANOVA with pairwise comparisons corrected for multiple comparisons was used with either a post hoc Tukey’s test or Sidak’s test.

For the comparison of P2Y1 receptor expression in CR cells, astrocytes, and pyramidal cells, correlation *R* among the three cell types was performed. Fisher’s transformation was applied to *R* so as to convert it to the *Z* distribution. After the conversion, a one-way ANOVA was performed (α = 0.05), with a post hoc Tukey test for multiple comparisons in order to compare between wild-type and *App^NL-F/NL-F^* mice in the three cell types: calretinin, astrocytes, and pyramidal cells.

For all statistical tests performed, a 95% confidence interval was used (*P* < 0.05).

The statistical analysis was performed using GraphPad Prism version 8.1.1 for Windows and Mac, GraphPad Software, La Jolla, CA, United States of America.

## Results

### CA1 Age-Dependent Phenotypical Changes in AD

**Figure 1:**
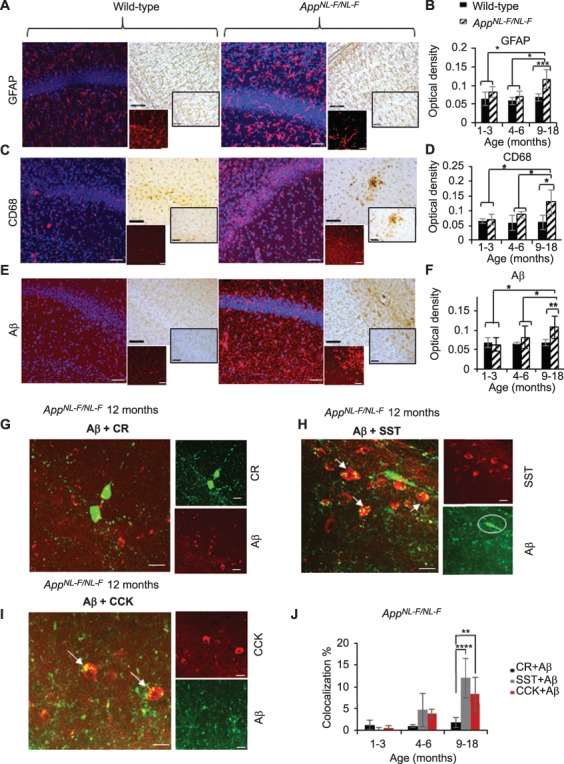
Age-dependent phenotypical changes in the *App^NL-F/NL-F^* model of AD. (*A*,*C*,*E*) *Z*-stack images from confocal microscopy illustrating the expression of GFAP (for reactive astrocytes), CD68 (for microglia), and Aβ (all in red, secondary antibody Texas Red) together with DAPI staining for nuclei (in blue) in 12-month age-matched wild-type and *App^NL-F/NL-F^* mice, respectively. Similarly, bright-field images of tissue immunostained with biotinylated antibodies show conglomerates of GFAP, CD68, and Aβ in the same animals. Both immunofluorescence and immunoperoxidase-stained images taken at ×20 magnification (larger images, scale bar = 50 μm) and ×63 magnification (inserts, scale bar = 20 μm). (*B*,*D*,*F*) Analysis of GFAP, CD68, and Aβ from immunoperoxidase-stained tissue. Significant differences in the three markers of AD were seen between wild-type and *App^NL-F/NL-F^* mice only at 9–18 months and when comparing quantification at 9–18 months with the other two age cohorts. (*G*,*I*) Age-dependent accumulation of Aβ in selective subtypes of interneurons in hippocampal CA1. Aβ colocalization was found at significantly higher levels in SST and CCK cells (indicated by arrows), but not in calretinin (CR) cells in the same animals at 12 months (scale = 20 μm). (*J*) Quantification of colocalization of Aβ with either CCK, SST, or calretinin cells. A two-way ANOVA was performed with pairwise comparisons corrected for multiple comparisons (α = 0.05), with either post hoc Sidak’s test or Tukey’s test for multiple comparisons. ^*^P < 0.05, ^**^P < 0.01, ^***^P < 0.001.

Classical hallmarks of AD, such as neuroinflammatory markers, astrocytes and microglia, and Aβ deposits, were stained with antibodies to glial fibrillary acidic protein (GFAP), CD68, and *APP*, respectively. Using immunofluorescence and immunoperoxidase staining, our data show that the *App^NL-F/NL-F^* model expresses an age-dependent accumulation of these classical hallmarks of AD (Fig. 1*A*–*F*). There was a significant change in glial cells and astrocytes and accumulation of Aβ levels only at 9–18 months between the age-matched wild-type and the *App^NL-F/NL-F^* mice. For example, GFAP levels significantly increased by 71% (±0.64%, *P* < 0.001, *n* = 10 animals per cohort, two-way ANOVA) (Fig. 1*A*,*B*) and CD68 levels increased by 108% (±27.71%, *P* < 0.05, *n* = 8 animals, two-way ANOVA) (Fig. 1*C*,*D*) in *App^NL-F/NL-F^* compared with the age-matched wild-type control mice at 9–18 months. There was also a 60% increase in overall Aβ accumulation in the entire CA1 region in the disease model compared with the age-matched wild-type mice at 9–18 months (±19.21%, *P* < 0.01, *n* = 8 animals, two-way ANOVA) (Fig. 1*E*,*F*). These differences were also significantly different when compared with the 1–3-month and 4–6-month age cohorts investigated (Fig. 1*B*,*D*, *F*).

### Aβ Accumulated in Specific Cell Types

To investigate whether Aβ selectively accumulated in specific cell populations, we used confocal microscopy to analyze the colocalization of Aβ with the somatic expression of either: CR-, SST-, or CCK-labeled cells in the *App^NL-F/NL-F^* mice. Interestingly, CR cells showed no significant colocalization with Aβ (in older animals, 1.4 ± 0.9% compared with wild-type mice, *P >* 0.5*, n* = 6 animals per group, two-way ANOVA) (Fig. 1*G*,*J*). In contrast, in the same AD mice, there was an age-dependent increase in the colocalization of Aβ levels with SST- and CCK-expressing cells in the *App^NL-F/NL-F^* mouse model (colocalization of SST with Aβ was 13 ± 4.0%, and CCK with Aβ was 9 ± 3.7%) (Fig. 1*H*–*J*), which were significantly different when compared with CR cell colocalization with SST/Aβ and CCK/Aβ at 9–18 months (*P* < 0.0001 for SST and *P* < 0.01 for CCK, *n* = 8 animals for both SST and CCK, two-way ANOVA), suggesting that CCK and SST cells are readily penetrated by Aβ.

### CCK- and SST-Expressing Interneurons Show an Age-Dependent Decline in AD, While CR Cells Remain Resilient

**Figure 2:**
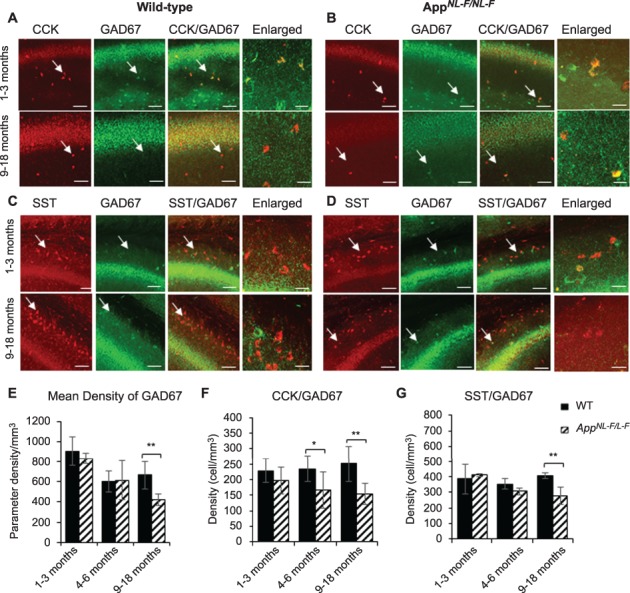
CCK- and SST-expressing interneurons show an age-dependent loss in the *App^NL-F/NL-F^* AD model. (*A*–*D*) Confocal microscope Z-stack images showing the expression of CCK- and SST-expressing cells (both in red channel), colocalized with GAD67, the marker for GABA production (green channel) in 1–3- and 9–18-month-old wild-type age-matched *App^NL-F/NL-F^* mice in CA1. Images taken at ×20 magnification (scale bar = 50 μm) and ×63 magnification (enlarged images, scale bar = 20 μm). In aged *App^NL-F/NL-F^* mice, CCK- and SST-positive cells were found to be weakly colocalized with GAD67 compared with the 1–3-month-old *App^NL-F/NL-F^* mice. (*E*–*G*) The graphs represent mean density of GAD67-, CCK-, and SST-positive cells in wild-type age-matched *App^NL-F/NL-F^* measured at three ages: 1–3 months, 4–6 months, and 9–18 months. Overall, GAD67, CCK, and SST expression showed an age-dependent decline in the AD model, which was significantly different from their control wild-type counterparts at 9–18 months. However, CCK cells also showed a significant decline in *App^NL-F/NL-F^* mice 4–6. A two-way ANOVA was performed with pairwise comparisons corrected for multiple comparisons (α = 0.05), with a post hoc Tukey’s test for multiple comparisons. ^*^P < 0.05, ^**^P < 0.001.

**Figure 3:**
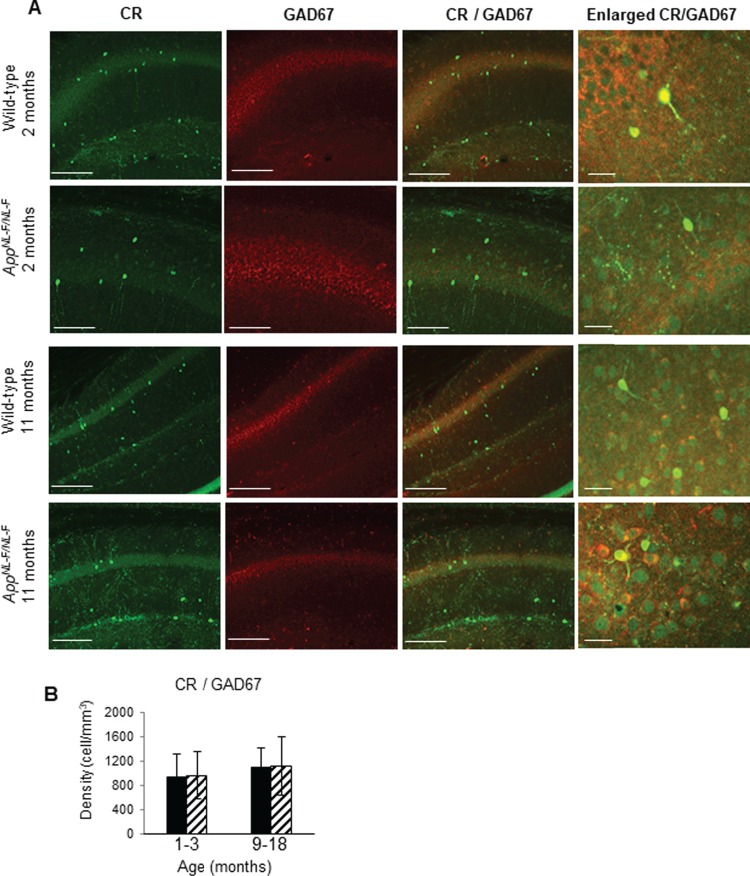
Calretinin (CR)-expressing interneurons are functionally and anatomically preserved in the CA1 in *App^NL-F/NL-F^* mice. (*A*) Z-stack confocal microscope images taken at ×20 magnification show calretinin (green, secondary antibody Alexa 488) and GAD67 (red, secondary antibody Texas Red), colocalized (yellow) in 2- and 12-month-old wild-type and *App^NL-F/NL-F^* mice. The nonsignificant change in the colocalization of GAD67- with CR-labeled cells between the two genotypes during the disease progression suggests that the integrity and function of the CR interneurons are preserved after postphenotypical alterations of the disease (scale bar = 50 μm). The enlarged images present wild-type and *App^NL-F/NL-F^* tissue imaged at ×63 magnification (scale bar = 20 μm). (*B*) Analysis of confocal images shows that the number of CR cells remains unchanged between wild-type and *App^NL-F/NL-F^* mice, in both young and old animals in CA1, CA2, and CA3 in the hippocampus. However, the amount of GAD67 appears to be increased significantly when colocalized with CR cells in 1–3-month-old *App^NL-F/NL-F^* mice when compared with age-matched wild-type mice. A two-way ANOVA (α = 0.05) was performed with pairwise comparisons corrected for multiple comparisons with Sidak’s test.

To determine whether general GABAergic inhibition was aberrant in the *App^NL-F/NL-F^* mouse model, we performed immunofluorescence studies to colocalize CCK-, SST-, and CR-expressing cells with glutamic acid decarboxylase 67 (GAD67), an enzyme for inhibitory neurotransmitter GABA production, which exists in every terminal where GABA locates and can be a reliable marker for functional GABAergic interneurons. Confocal *Z*-stack images showed a general age-dependent decline of GAD67 (Fig. 2*E*), which was significantly decreased in the oldest cohort of *App^NL-F/NL-F^* mice studied (1–3 months, 7.73% ± 1.20%, *P* > 0.05, *n = 5,* and at 9–18 months, 36.09% ± 2.07, *P <* 0.01, *n = 5*, two-way ANOVA). This suggests that GABAergic cells maintained their function in the early stages of the disease, but this altered as the pathology of the disease progressed, which is consistent with previous studies using alternative AD mouse models (Krantic et al. 2012; Choi et al. 2018; Leung et al. 2012). We then measured the expression of CCK, SST, and CR colocalization with GAD67 (Figs 2 and 3) from *Z*-stack images obtained using confocal microscopy. All somata labeled with respective neuropeptide (CCK, SST) or calcium-binding protein (CR) always colocalized GAD67, although there was a cell-type specific alteration in the expression with age in the AD model compared with the age-matched wild-type mice. For example, a significant decline in the expression of the CCK/GAD67-labeled cells was observed, as well as a decrease in CCK cells from 4 months onwards in the *App*^NL-F/NL-F^ mice and a decrease of 24.31 ± 1.39 (*P <* 0.05, *n =* 4, two-way ANOVA) at 4–6 months and 35.91 ± 3.10% (*P* < 0.01, *n = 7, t*-test) at 9–18 months (Fig. 2*F*). Similarly, SST/GAD67 cells also significantly decreased in an age-dependent manner in the *App^NL-F/NL-F^* mouse model, and there was a reduction of 15.44 ± 2.94 (*P* > 0.05, *n* = 4, two-way ANOVA) and a significant reduction of 32.02 ± 2.65% (*P* < 0.01, *n = 8* for wild-type, *n = 5 App^NL-F/NL-F^*, two-way ANOVA) at 4–6 months and 9–18 months, respectively, in the *App^NL-F/NL-F^* mice age-matched to control wild-type mice (Fig. 2*G*). In contrast to the SST-expressing and CCK-expressing interneurons, in the *App^NL-F/NL-F^* mouse model, we discovered a preservation of the CR-expressing interneurons colocalized with GAD67 in all the age-matched wild-type and *App^NL-F/NL-F^* animals studied (Fig. 3*A*,*B*).

**Figure 4:**
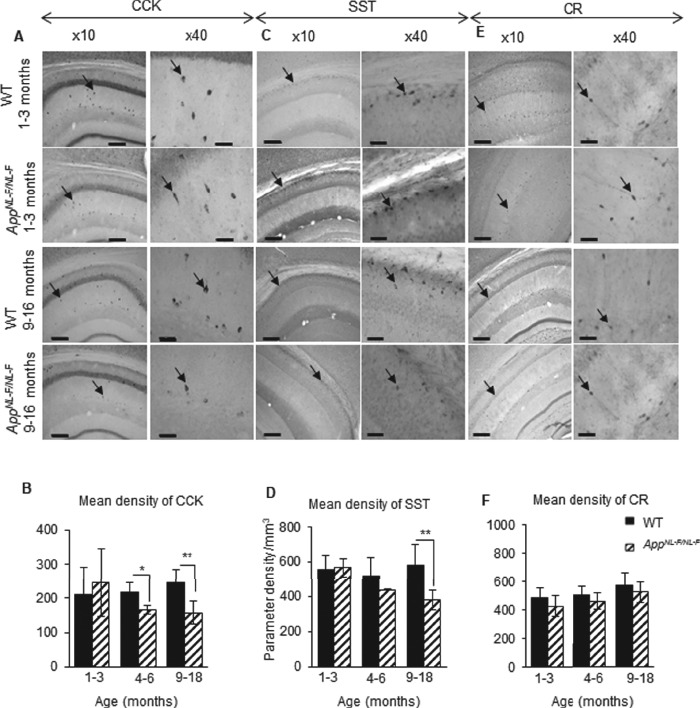
Cell-type–specific survival of CA1 interneurons in the *App^NL-F/NL-F^* AD model. (*A*–*C*) Immunoperoxidase staining showing CCK, SST, and CR interneurons, respectively, at ×10 and ×40 magnification in age-matched wild-type and *App^NL-F/NL-F^* animals (arrows indicate example interneurons). Scale bar, 100 μm for ×10 images and 50 μm for ×40. (*D*–*F*) Graphs illustrate a significant decrease in SST cells in the 9–18-month-old *App^NL-F/NL-F^* animals compared with the age-matched wild-type cohort. There was also a significant decrease in CCK cells at 4–6-month-old and 9–18-month-old *App^NL-F/NL-F^* animals compared with age-matched wild-type animals. In contrast, CR cell densities were not significantly different between the ages and genotypes studied. A two-way ANOVA (α = 0.05) was performed with pairwise comparisons corrected for multiple comparisons with Sidak’s test. ^*^P < 0.05, ^**^P < 0.01.

The changes in the cell densities were also corroborated by immune peroxidase staining, which showed a similar alteration in the densities of CCK- and SST-expressing cells in CA1 of *App^NL-F/NL-F^* mice age-matched to wild-type mice (Fig 4*A*–*D*), while no significant differences in the CR cell density between *App^NL-F/NL-F^* mice and age-matched to wild-type control were seen at any of the age cohorts (Fig. 4*E*,*F*). A nonsignificant increase of 2–3% was observed in the AD model age-matched to the wild-type mice (*P* > 0.5, *n* = 9, two-way ANOVA).

### Membrane Properties of CCK and SST Interneurons Are Hyperactive While CR Cells Are Preserved in Early Stages of AD

To identify contributing factors to the loss of cell densities, we investigated the cell membrane properties of CCK, SST, and CR cells at between 1.5 and 2 months of age in both genotypes to detect possible differences preceding the classical hallmarks of AD such as Aβ accumulation and neuroinflammation. Due to the lack of identifiable CCK and SST somata under infrared-differential interference contrast (IR-DIC) in the late stages of AD (9–18 months), it was not possible in our hands to record CCK and SST cells in the older cohorts.

**Figure 5:**
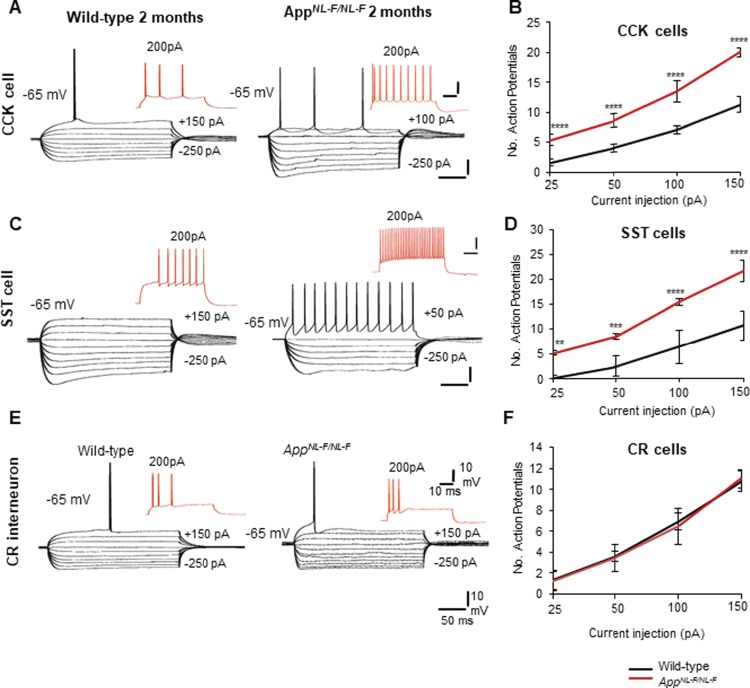
CCK and SST cells displayed hyperactive membrane properties, but CR cells remained unchanged in early AD. (*A*,*C*) Intrinsic membrane response of CA1 CCK interneurons and SST interneurons recorded using whole-cell patch clamp electrodes in wild-type and *App^NL-F/NL-F^* mice at 2 months of age. Both CCK and SST cells displayed hyperexcitable membranes at −65 mV in response to intracellular current injections (ranging from +200 to −200 pA). Red traces are the voltage response of the cells to +200 pA current injection. (*B*,*D*) Graphs illustrate the number of action potentials discharged with increasing current applied to CCK and SST cells recorded in wild-type and *App^NL-F/NL-F^* mice. The firing of both interneurons was dramatically increased with increasing current injections in the AD model, which was also accompanied by an increase in input resistance and time-constant illustrating hyperexcitability in the AD model. (*E*) Intrinsic membrane response of CA1 CR interneurons recorded in 2-month wild-type and *App^NL-F/NL-F^* mice, respectively. These showed passive responses to intracellular current injections (+200 to −200 pA) which culminated in single- or double-action potentials with current injection of +150 pA (black traces). Red traces are the voltage response of the cells to +200-pA current injection. There were no significant differences in the action potential discharge, input resistance, and time constants between the two age-matched mouse cohorts. (*F*) The input–output curves displayed a pseudolinear relationship between number of action potentials generated by adapting CR cells of healthy wild-type and *App^NL-F/NL-F^* mice with increasing current injections. The membrane input resistance, action potential threshold, and time constants did not appear to be different between the two genotypes studied. A two-way ANOVA was performed with genotype and treatment as factors (α = 0.05), with a post hoc Sidak’s test for multiple comparisons. ^*^P < 0.05, ^**^P < 0.01, ^***^P < 0.001, ^****^P < 0.0001.

Recorded cells were initially identified by their somata location in CA1 and electrophysiological properties, followed by their gross morphology (post recording). Morphologically, CCK cells had triangular somata located in the stratum radiatum (SR) with dendrites predominantly radiating in SR. The axons of these cells ramified in SR with a few branches extending into the stratum pyramidale (SP) (see Ali 2007). However, the SST cell somata located in the stratum oriens (SO) had horizontally oriented dendrites restricted to SO, and the axons of these cells projected and ramified extensively in the stratum lacunosum moleculare (SLM). Furthermore, SST cells displayed a characteristic “sag” in the electronic response to hyperpolarizing current (Fig. 5*C*) characterization of *I*_h_ current activation in the cells (see also Ali et al. 1998).

Interestingly, the membrane properties of CCK interneurons were consistently found to be intrinsically hyperactive (Fig. 5*A*,*B*; see Table 2 for details). Similarly, SST cell membrane properties were also hyperexcited (Fig. 5*C*,*D*), which was consistent with previous studies (Zhang et al. 2016). The membrane hyperexcitability was indicated by their decreased firing threshold which was accompanied by an increased membrane input resistance, time constant, and action potential firing frequency in *App^NL-F/NL-F^* mice compared with the age-matched control wild-type mice with the same magnitude of current injection at the same membrane potential (Fig. 5*B*,*D* and Table 2).

**Table 2:** Electrophysiological properties of CCK, SST, and CR interneurons recorded from wild-type age-matched to *App^NL-F/NL-F^* mice in the CA1 region of the hippocampus

Subclass of cells	**CCK** (*n* = 20 cells, 6 animals per genotype)		SST (*n* = 10 cells, 5 animals per genotype)		CR cells (*n* = 14 cells, 8 animals per genotype)		CR cells (*n* = 12 cells, 8 animals per genotype)	
	Wild-type	*App^NL-F/NL-F^*	Wild-type	*App^NL-F/NL-F^*	Wild-type	*App^NL-F/NL-F^*	Wild-type	*App^NL-F/NL-F^*
AP Amp (mV)	78 ± 2.50	68 ± 2.00	82 ± 3.00	70 ± 5.40	78 ± 1.50	78 ± 2.00	76 ± 1.50	78 ± 2.00
AP HW (ms)	1.3 ± 0.43	1.5 ± 0.320	1.2 ± 0.25	1.5 ± 0.50	1.25 ± 0.32	1.3 ± 0.22	1.25 ± 0.32	1.3 ± 0.22
AP threshold (mV)	22 ± 1.56	17.5 ± 1.30^*^	20 ± 1.50	14.5 ± 1.32^*^	22 ± 1.43	22 ± 1.47	22 ± 1.43	22 ± 1.47
AP AHP Amp (mV)	6.5 ± 0.75	4.37 ± 0.90	8.7 ± 0.63	5.50 ± 0.74	9.4 ± 0.65	9.56 ± 0.67	9.4 ± 0.65	9.56 ± 0.67
Input resistance (MΩ)	295 ± 14.52	350.45 ± 18.10^**^	300.54 ± 25.0	365.53 ± 30.56^**^	290 ± 23.00	291 ± 36.64	290 ± 23.00	291 ± 36.64
TC (ms)	12.0 ± 2.00	15.70 ± 0.62^*^	10.52 ± 1.42	16.47 ± 0.95^**^	10.50 ± 0.50	10.32 ± 0.50	10.50 ± 0.50	10.32 ± 0.50
No. of spikes at +150 pA	11 ± 1.30	21 ± 0.37^****^	10.75 ± 2.98	21.75 ± 2.21^****^	10.75 ± 0.95	11 ± 0.81	11.20 ± 0.85	10.95 ± 0.94
1.5–2 months			1.5–2 months		1.5–2 months		9–18 months	

Results are expressed as mean ± SD. A two-sampled unpaired Student’s *t*-test was used to compare between control wild-type and *App^NL-F/NL-F^* mice for each biophysical property per cell type.

AP, action potential; Amp, amplitude; HW, half-width; AHP, after hyperpolarization; TC, time constant.

Significantly different from control, unpaired *t*-test, ^*^P < 0.05, ^**^P < 0.01, ^****^P < 0.0001.

**Figure 6:**
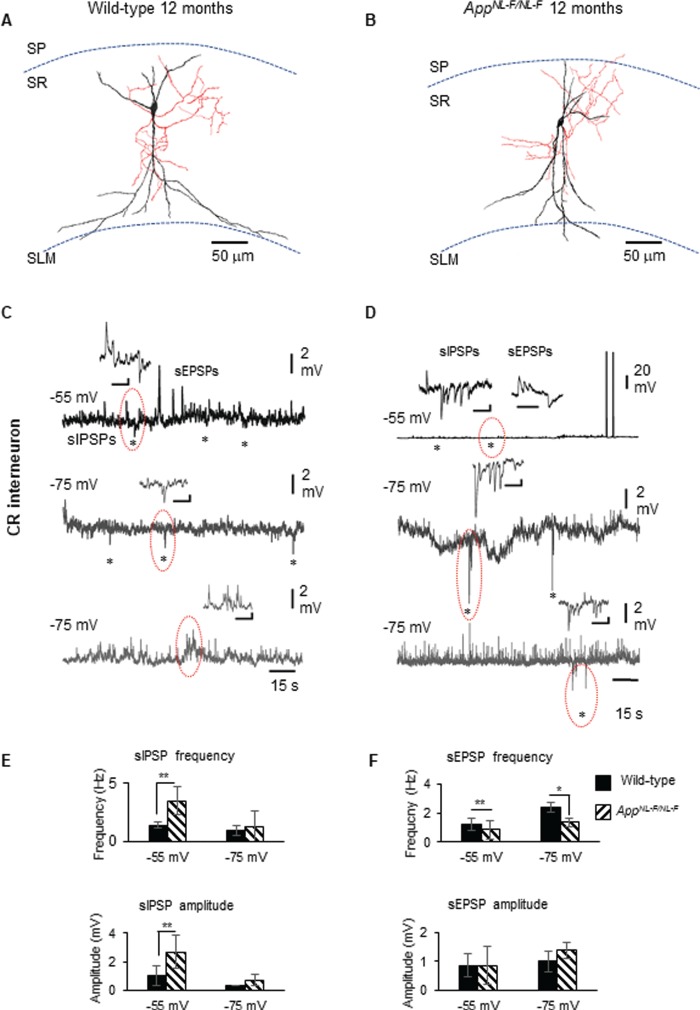
Calretinin cells in early AD form an enhanced inhibitory network. (*A*,*B*) Reconstruction of recorded, biocytin-labeled CR cells from wild-type mouse and *App^NL-F/NL-F^* mouse model, drawn at ×1000 magnification using a light microscope and drawing tube. The dendrites (black) and axon (red) were located in SR. The morphology of CR cells in both genotypes was similar in appearance. (*C*,*D*) Whole-cell current-clamp recordings of spontaneous inhibitory/excitatory postsynaptic potentials (sIPSPs and sEPSPs) recorded in CR cells in CA1 of 12-month-old *App^NL-F/NL-F^* mice, at membrane potentials of −55 and − 75 mV in control conditions (morphology shown above). The circles indicate where synaptic events have been enlarged and shown in the inserts. (*E*,*F*) Bar graphs show the average sIPSP and sEPSP amplitude and frequency at −55 and − 75 mV in CR cells recorded in wild-type and *App^NL-F/NL-F^* mouse models. These data suggest a significantly enhanced frequency and amplitude of inhibition at −55 mV, while sEPSP frequency (and not amplitude) was found significantly different at both −55 and − 75 mV recorded in CR cells at 9–18 months of age in *App^NL-F/NL-F^* mice. A two-way ANOVA was performed with pairwise comparisons corrected for multiple comparisons (α = 0.05), with a post hoc Tukey’s test for multiple comparisons. ^*^P < 0.05, ^**^P < 0.01, ^***^P < 0.001, ^****^P < 0.0001.

In contrast to intrinsic hyperactivity observed in CCK and SST cells, the intrinsic membrane properties of CR cells were found to be unchanged at a younger age range (1.5–2 months) (Fig. 5*E*,*F*) between wild-type age-matched to *App^NL-F/NL-F^* mice. Furthermore, the CR cells recorded at the later stage of 9–18 months also remained unchanged between the two genotypes (Table 2). The membrane properties of CR cells—input resistance, time constants, and action potential firing properties—remained unchanged during the disease progression (Fig. 5*F*). Morphologically, the recorded CR cells were similar in appearance (Fig. 6*A*,*B*), and in our data sets all had oval somata located in mid SR with two to three primary dendrites that radiated into SR and never entered SP. The axons of CR cells were sparse and ramified close to the somata in SR; these cells resembled previously published CR cells (see Gulyas et al. 1996).

### CR Interneurons Displayed Strengthened Synaptic Inhibition in AD

Spontaneous inhibitory postsynaptic potentials (sIPSPs) and spontaneous excitatory postsynaptic potentials (sEPSPs) were recorded from CR interneurons in 1–3- and 9–18-month old *App^NL-F/NL-F^* mice at holding membrane potentials of −55 and −75 mV (Fig. 6*C*–*F*); however, no differences were observed in the synaptic properties received by CR cells at the two age cohorts studied in either genotypes. Since our aim was to investigate whether CR cells were functionally “preserved” during the presence of significant Aβ accumulation and proliferation of glial cells and astrocytes, we have presented the data sets obtained “post phenotypic changes” of AD (9–18 months), which revealed interesting properties compared with the age-matched wild-type CR cells recorded.

The average peak frequency and amplitude of sIPSPs increased in the AD model compared with wild-type age-matched mice at both membrane potentials, but were only significantly different at a more positive membrane potential of −55 mV (due to the higher imposed driving force for Cl^−^ ions to enter GABA_A_ receptors in our experimental condition). In the *App^NL-F/NL-F^* mice, sIPSP frequency and amplitude increased by 153 ± 53% (*P* < 0.01, *n* = 6, two-way ANOVA) and 157 ± 65% (*P* < 0.01, *n* = 6, two-way ANOVA) of control sIPSPs recorded in age-matched wild-type mice, respectively (Fig. 6*E*). The average sEPSP frequency also changed significantly, showing a decrease at both membrane potentials, −55 and − 75 mV, but without a significant change in the amplitudes (Fig. 6*F*). The increase in sEPSP frequency was 144 ± 35% (*P* < 0.01, *n* = 6, two-way ANOVA) and 87 ± 19% (*P* > 0.05, *n* = 6, two-way ANOVA), of control wild-type sEPSPs recorded at −55 and − 75 mV, respectively.

CR interneurons during the late stages (9–18 months) of AD were readily identifiable under IR-DIC during experiments, which was in striking contrast to CCK or SST cells that were not easily visualized. These differences could be due to the decline of CCK and SST cells in the late stages of the disease; this together with the technical difficulties of performing whole-cell recordings in aged mice hampered recording of CCK and SST cells in these animals to directly compare the synaptic inputs of CCK and SST cells after postphenotypical alterations of AD.

### P2Y1Rs Are Expressed Predominantly in Calretinin Cells

**Figure 7:**
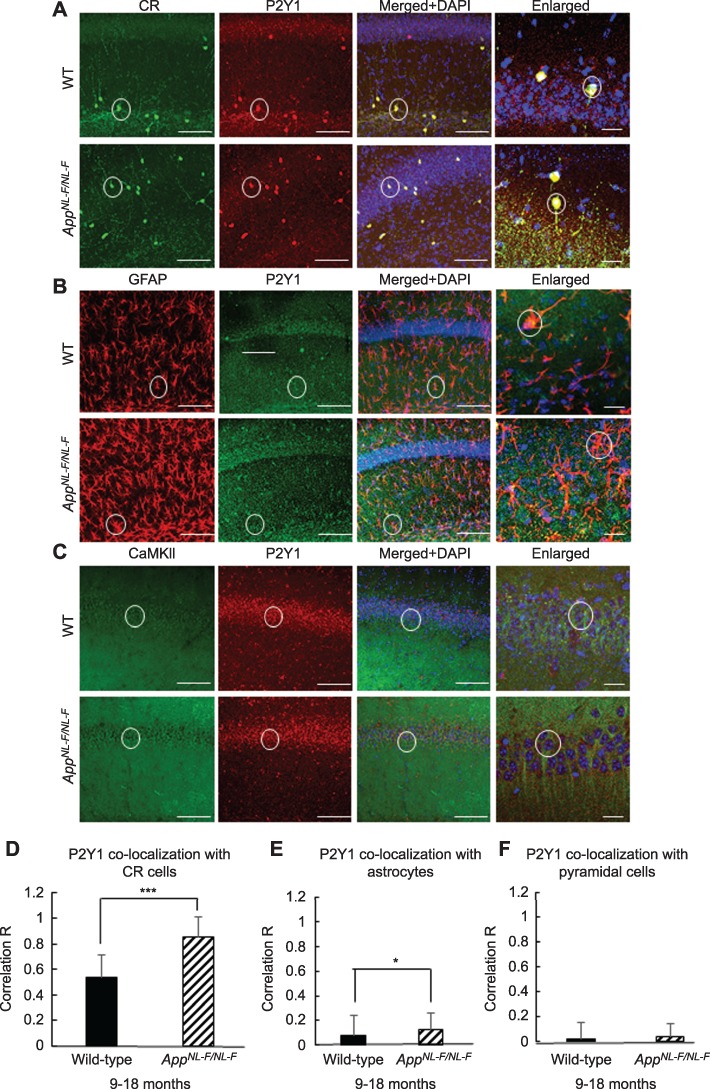
P2Y1Rs are predominantly expressed on CR cells and are upregulated in the *App^NL-F/NL-F^* AD mouse model. (*A*–*C*) Confocal microscope, Z-stack images of P2Y1R colocalization on CR cells (green, Alexa 488), astrocytes stained for GFAP (red, Texas Red), and pyramidal cells stained for CaMKII-α (green, FITC), respectively in 9–18-month-old wild-type and *App^NL-F/NL-F^* mice (taken at ×63 magnification, scale bar, 20 μm). Representative cells are outlined with white circles, and colocalization between the two channels appears yellow/orange. The merged images include the nuclear staining, DAPI (blue). (*D*–*F*) Graphs illustrate quantification of P2Y1Rs colocalized on CR cells, GFAP, and CaMKII-α in wild-type and *App^NL-F/NL-F^* mice obtained from *Z*-stack images at ×20 magnification. The CR cells and astrocytes show a high level of coexpression with P2Y1Rs; however, the CR cells showed the highest level of P2Y1R coexpression, which seems to be upregulated in the AD model. Error bars represent the standard deviation from the mean. Two-tailed unpaired Student’s *t*-test was performed individually for panels (*D*–*F*) to compare wild-type and *App^NL-F/NL-F^* mice within their respective groups. To measure across the three cell groups studied, a one-way ANOVA was performed with pairwise comparisons corrected for multiple comparisons (α = 0.05), with a post hoc Tukey’s test for multiple comparisons. ^*^P < 0.05, ^**^P < 0.01, ^***^P < 0.001 (40–240 cells, *n* = 4 animals for CR cells and *n* = 6 animals for GFAP and CaMKII-α).

Previously, it has been shown that P2Y1Rs are expressed on CR cells (Bowser and Khakh 2004), and others have also evidenced that these receptors are “upregulated” in proinflammatory reactive astrocytes in AD, which facilitates the synchrony of aberrant astrocyte behavior (Delekate et al. 2014). Whether similar mechanisms exist among the CR cell networks that promote hyperinhibition in AD is unknown, and therefore, we investigated the level of expression colocalization of P2Y1Rs on CR cells and compared the level of P2Y1R colocalization to either GFAP (for reactive astrocytes) or CaMKII-α (for pyramidal cells) from the oldest cohort (after phenotypic changes of the disease), 9–18-month-old *App^NL-F/NL-F^* and wild-type mice (Fig. 7*A*–*C*). We investigated the colocalization of P2Y1Rs which was estimated using the correlation coefficient *R* to assess the level of correlation between the fluorophore channels that correspond to either CR, GFAP, or CaMKII-α (see Methods).

We found a high level of colocalization of P2Y1Rs in CR cells compared with astrocytes and pyramidal cells in 9–18-months age-matched wild-type and *App^NL-F/NL-F^* mice (Fig. 7*D*–*F*). In wild-type mice, a 13- and 23-fold increase in P2Y1 receptor colocalization in CR cells was observed compared with P2Y1 colocalization in GFAP and CaMKII-α-labeled cells, respectively (*P* < 0.05, *n* = 4 animals for CR cells, and *n* = 6 animals for GFAP and CaMKII-α labeled cells). In *App^NL-F/NL-F^* mice, there was an 8- and 16-fold increase in the colocalization of the P2Y1 receptor on CR cells compared with P2Y1 receptor colocalization in GFAP and CaMKII-α-labeled cells, respectively (*P* < 0.05, *n* = 6 animals). Furthermore, there was an upregulation of P2Y1R expression in the AD model, which was correlated with a higher colocalization in CR cells, astrocytes, and pyramidal cells of *App^NL-F/NL-F^* mice compared with the age-matched wild type. For example, there was a significant increase in the receptor coexpressed with CR cells in the *App^NL-F/NL-F^* mice compared with the age-matched wild-type CR cells (28.37 ± 0.41% of control wild-type, *P* < 0.001, *n* = 40 cells, *n* = 4 animals per group). In the *App^NL-F/NL-F^* mice, there was also an increase in the expression of P2Y1Rs with GFAP by 102.61 ± 113.35% of control (*P* < 0.05, *n* = 240 cells, *n* = 6 animals per group) and CaMKII-α by 77.6 ± 55.90% of control wild type (*P* > 0.05, *n* = 60 cells, *n* = 6 animals per group).

### P2Y1 Receptor Allosteric Inhibitor Restores Dysfunctional Inhibitory Homeostasis in CA1

Our data suggests an overall increase in the level of P2Y1R expression in the AD model; therefore, we investigated whether blocking P2Y1Rs *in vitro* could “normalize” the hyperinhibition observed in CR cells. Using *App^NL-F/NL-F^* knock-in mice and age- and sex-matched wild-type controls, we bath-applied the P2Y1 receptor agonist, MRS2365 (500 nM), followed by subsequent addition of the P2Y1R allosteric inhibitor, BPTU (500 nM). Since we previously reported that principal pyramidal cells were aberrantly hyperexcited following hypoinhibition input (Petrache et al. 2019), it was of interest to also investigate the changes in synaptic activity recorded in pyramidal cells after bath application of the P2Y1R modulators.

**Figure 8:**
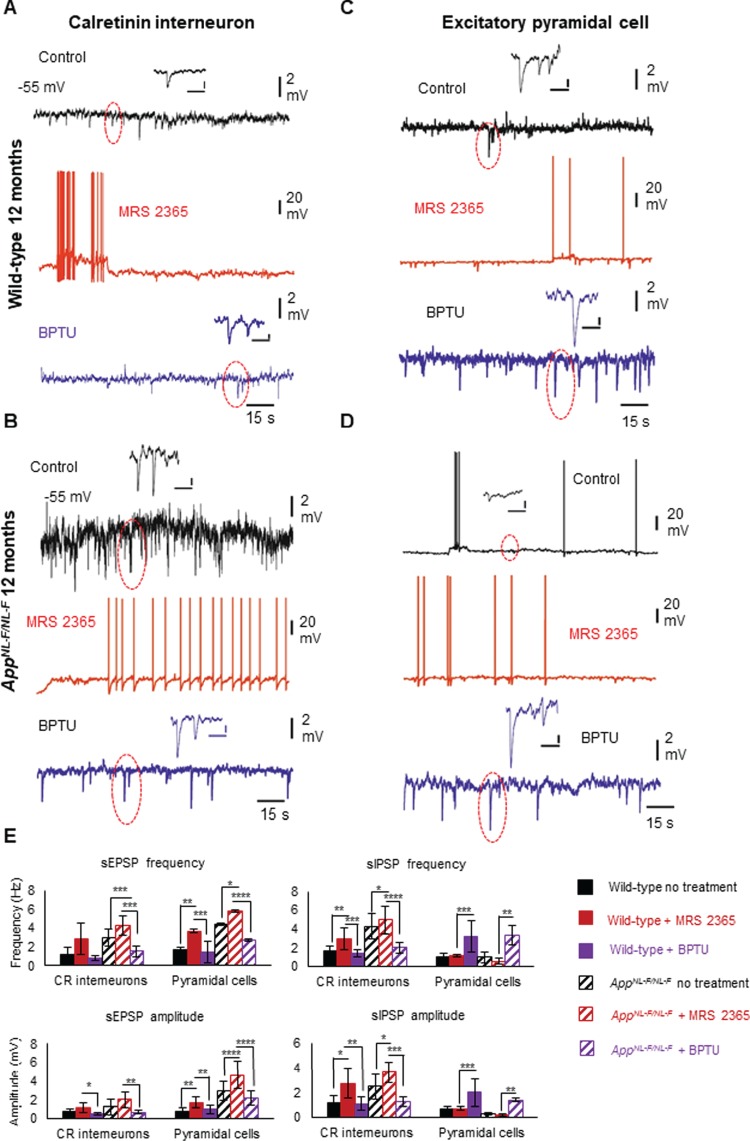
Enhanced inhibition among CR interneuron networks in AD is modulated by upregulated P2Y1Rs. (*A*–*D*) Whole-cell current-clamp recordings illustrating sIPSPs and sEPSPs in CR cells (*A*, *B*) and pyramidal cells (*C*, *D*) in CA1 of 12-month-old wild-type and *App^NL-F/NL-F^* mice, recorded at a membrane potential of −55 mV in control conditions and after bath application of the P2Y1R modulators. Bath application of the P2Y1R-selective agonist, MRS2365 (500 nM), resulted in membrane excitation in both cell types, increasing the aberrant hyperexcitability. However, subsequent addition of the P2Y1R inhibitor BPTU (500 nM) differentially affected the cell types; the sIPSPs (examples are highlighted in circles) were dampened in CR cells, but increased in pyramidal cells, thus normalizing the homeostatic levels of inhibition in CA1. Similar results were obtained from wild-type mice shown in bar graphs. (*E*) Bar graphs show the overall pharmacological data obtained from wild-type and *App^NL-F/NL-F^* mice at 9–18 months. A two-way ANOVA was performed with pairwise comparisons corrected for multiple comparisons (α = 0.05), with a post hoc Tukey’s test for multiple comparisons. ^*^P < 0.05, ^**^P < 0.01, ^***^P < 0.001, ^****^P < 0.0001. (see Table 3 for details).

In both wild-type and *App^NL-F/NL-F^* mice, bath application of MRS2365 resulted in an enhanced aberrant hyperactivity in both CR and pyramidal cells recorded at −55 mV by causing an increase in action potential discharge, an increase in sEPSP amplitude and frequency (see Table 3 for details) (Fig. 8), and an average ~ 8-mV depolarization of the cell membrane. Subsequent addition of the inhibitor BPTU “decreased” the hyperinhibition observed in CR cells by decreasing the MRS2365-induced firing but also significantly reducing the sIPSP amplitudes and frequency in CR cells (Table 3 and Fig. 8*A*,*B*,*E*). However, the aberrant hyperexcitability of principal pyramidal cells was differentially affected in comparison with CR interneurons following application of P2Y1R inhibitor BPTU. Upon subsequent bath application of BPTU, there was an increase in the sIPSP amplitude and frequency (Table 3) (Fig. 8*C*–*E*), with an ~ 10-mV tonic hyperpolarization of pyramidal cell membranes. Thus, BPTU produced a normalization of the aberrant hyperinhibition at CR cells and consequently the aberrant hypoinhibition at pyramidal cells in the AD model.

**Table 3:** P2Y1 receptor pharmacology in CR and pyramidal cells in 9–18-month wild-type and *App^NL-F/NL-F^* mice

	Calretinin cells		Pyramidal cells	
sEPSP frequency				
Genotype	MRS2365 (% change from control)	BPTU (% change from MRS2365)	MRS2365 (% change from control)	BPTU (% change from MRS2365)
Wild-type	↑131.72 ± 77.80 (*n* = 5)	↓ 69.64 ± 20.89 (*n* = 5)	↑ 110.38 ± 4.45 (*n* = 6)^**^	↓ 61.14 ± 47.93 (*n* = 6)^***^
*APP^NL-F/NL-F^*	↑ 44.07 ± 10.24 (*n* = 6)^***^	↓ 64.31 ± 23.90 (*n* = 6)^***^	↑ 32.57 ± 0.44 (*n* = 5)^*^	↓ 53.45 ± 2.80 (*n* = 5)^****^
sEPSP amplitude				
Wild-type	↑ 53.91 ± 23.39 (*n* = 5)	↓ 56.44 ± 8.49 (*n* = 5)^*^	↑ 121.51 ± 39.96 (*n* = 6) ^**^	↓ 42.72 ± 18.52 (*n* = 6)^**^
*APP^NL-F/NL-F^*	↑ 62.16 ± 26.42 (*n* = 5)	↓ 65.42 ± 25.21 (*n* = 5)^**^	↑ 59.48 ± 19.03 (*n* = 5)^****^	↓ 53.51 ± 19.12 (*n* = 5)^****^
sIPSP frequency				
Wild-type	↑ 80.98 ± 30.46 (*n* = 6)^**^	↓ 52.54 ± 15.01 (*n* = 6)^***^	↑ 13.93 ± 2.10 (*n* = 6)	↑ 176.26 ± 91.11 (*n* = 6)^***^
*APP^NL-F/NL-F^*	↑ 16.64 ± 4.95 (*n* = 7)^*^	↓ 59.37 ± 17.47 (*n* = 7)^****^	↓ 40.25 ± 20.24 (*n* = 5)	↑ 465.96 ± 143.54 (*n* = 5)^**^
sIPSP amplitude				
Wild-type	↑ 132.29 ± 50.57 (*n* = 6)^*^	↓ 60.0 ± 29.07 (*n* = 6)^**^	↑ 121.51 ± 39.96 (*n* = 6) #8	↑ 42.72 ± 18.52 (*n* = 6)^***^
*APP^NL-F/NL-F^*	↑ 45.98 ± 10.21 (*n* = 7)^*^	↓ 64.76 ± 17.90 (*n* = 7)^***^	↑ 59.48 ± 19.03 (*n* = 5)	↑ 53.51 ± 19.12 (*n* = 5)^**^

Changes of spontaneous synaptic events recorded in CR and pyramidal cells after bath application of the P2Y1R agonist, MRS2365, followed by antagonist, BPTU, in 9–18 months of age-matched, wild-type, and *APP^NL-F/NL-F^* mice. Values represent % changes between conditions (MRS2365 application after control or BPTU after subsequent addition of MRS2365 to control) ± standard deviation. A one-way ANOVA followed by post hoc Tukey’s test for multiple comparisons was used to determine the statistical value. Sample size *n* denotes the number of animals (one cell per animal was recorded in these experiments). Arrows indicate either a decrease or increase in the parameter. MRS2365, P2Y1 agonist; BPTU, P2Y1 allosteric antagonist.

^*^ *P* < 0.05, ^**^P < 0.01, ^***^P < 0.001, ^****^P < 0.0001.

## Discussion

**Figure 9:**
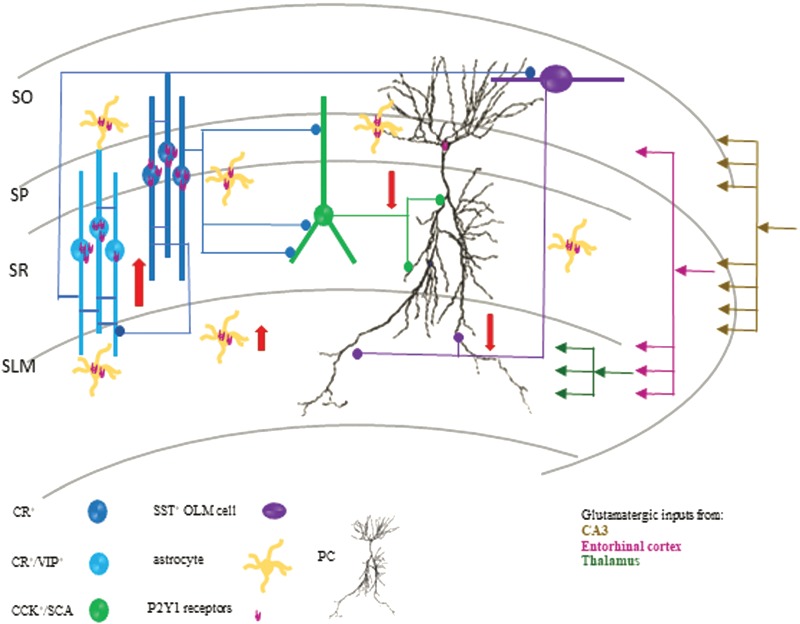
Schematic diagram of a CA1 circuit involving the major modulatory interneuron circuits and excitatory pyramidal cells in late AD. Red arrows depict the increase in CR cell inhibitory function and a decrease in CCK- and SST-expressing interneuron function in CA1. It has been evidenced that CCK cells fine-tune proximal pyramidal cells, while SST-expressing cells fine-tune distal dendrites of pyramidal cells; therefore, loss of their function from ~ 6 months will impact on CA1 pyramidal cell excitability and promote pyramidal cell hyperexcitability (due to hypoinhibition). We suggest that the early hyperactivity of CCK and SST cells facilitates increased Αβ cleavage and accumulation that subsequently leads to their destruction. These factors, in addition to the overactivity among CR cells in the later stages of the disease, leads to deficits in pyramidal cell inhibition. Furthermore, we suggest that the enhanced hyperinhibition observed in CR cells is partly due to hypertrophy of astrocytes and upregulated P2Y1Rs predominantly coexpressed on CR cells and astrocytes, which is restored by blocking upregulated P2Y1Rs.

Using the first knock-in mouse model of AD (*App^NL-F/NL-F^)* (Saito et al. 2014), we showed an age-dependent increase in the pathological hallmarks of AD, including Αβ and microglial and reactive astrocytes, and several novel observations in the CA1 region of the hippocampus, which are illustrated in the schematic circuit diagram in Figure 9. Firstly, we report a cell-type–specific alteration of modulatory interneuron function and association with Aβ oligomers. There was a gradual decline in the expression of CCK- and SST-expressing inhibitory interneurons together with the coexpression of GAD67, suggesting a reduction in their inhibitory function. The decrease in SST expression is consistent with the results that SST expression decreases in the cortex and hippocampus in AD patients (Liguzlecznar et al. 2016), as was the age-dependent decline in GAD67 (Krantic et al. 2012; Choi et al. 2018; Leung et al. 2012). The CCK and SST cells also colocalized a significant amount of Aβ fragments, and their biophysical properties showed aberrant hyperexcitability in the early stages of AD. In striking contrast, the density of CR cells and coexpression of GAD67 were unchanged in our AD model, consistent with anatomical studies reporting a resilience of CR cells in postmortem brains of AD patients (Fonseca and Soriano 1995). Furthermore, we demonstrate that the CR cells did not colocalize Aβ fragments and maintained their intrinsic biophysical properties, showing a surprising resilience to alteration during the pathobiology of AD in the *App^NL-F/NL-F^* model.

Our second key finding is related to alteration in inhibitory synaptic activity. We observed an enhanced frequency and amplitude of spontaneous synaptic inhibition among the CR cells proceeding the pathological hallmarks of AD, which suggests changes in pre- and postsynaptic factors during the pathogenesis of the disease. This enhanced inhibitory activity in CR cells in the AD model was despite a hyperexcited state of excitatory pyramidal cells. Others have also shown in a transgenic *App* mouse model of AD that pathological seizure-like activity in the cortex and hippocampus is accompanied by enhanced “compensatory” inhibitory activity (Palop et al. 2007). We demonstrate that CR cells together with astrocytes express a high level of P2Y1Rs in comparison to principal pyramidal cells in CA1, although we do not exclude the possibility that P2Y1R expression is changed in other cell types. Finally, we show that blockade of P2Y1Rs via allosteric inhibition restored inhibition to “normal” at CR interneurons and, as a consequence, corrected also the hyperexcitability of pyramidal cells to normal synaptic levels.

### Selective Association of Αβ in CA1 Interneurons

The selective colocalization of Αβ peptides is correlated with the intrinsic hyperactivity of CCK and SST cells reported in our study in the early-stage, preplaque formation of AD, but in the presence of a low level of soluble Αβ peptides, probably with high Αβ 42/40 ratio. This is consistent with other studies (Shah et al. 2018; Palop and Mucke 2016a). Whether the hyperexcitability of CCK and SST cells results in Αβ production or whether Αβ infiltration in the cells results in the hyperexcited state is yet to be fully explored. Interestingly, both SST peptide and APP undergo similar cleavage processes to form SST and Aβ, which could facilitate interactions between the two even before their release from cells (LaFerla et al. 2007), which has led to the SST neuropeptide being termed “amyloidogenic.” Consistent with the aforementioned findings, SST has also been shown to be one of the neuropeptides that bind to Aβ fragments in vitro, enabling their oligomerization to amyloidogenic peptide (Solarski et al. 2018), and SST cells in the piriform cortex and olfactory cortex are found highly colocalized with Aβ (Saiz-Sanchez et al. 2015). Furthermore, SST is thought to regulate Aβ degradation by modulating neprilysin which is an essential protein for degrading Aβ (Saito et al. 2005). Perhaps it is these common cleavage mechanisms in combination with toxic soluble Αβ infiltration that makes SST interneurons vulnerable to Aβ infiltration that triggers the aggregation and degradation of Aβ resulting in the high-level intracellular Aβ in SST. This probably destroys the normal function of SST cells and results in cell death. Whether similar mechanisms exist for CCK cells that also colocalized high Aβ needs investigation; however, it is possible that APP and Aβ are part of a feedback loop that controls the intrinsic neuronal excitability (Kamenetz et al. 2003) which we observe in both SST and CCK cells.

The selective protection from Aβ penetration could also be related to the neurochemical and pharmacological profile of the cell type since, in our study, CR cells were preserved anatomically and physiologically. Calretinin is a calcium-binding protein parvalbumin (PV), and cells expressing this protein are also reported to be either preserved, increased, or decreased, depending on the cortical region reported in various models of AD from early to late stages of the disease (Yang et al. 2018; Zallo et al. 2018; Petrache et al. 2019) which could be associated with calcium homeostasis. In normal conditions, Ca^2+^ is able to regulate the cellular membrane properties via voltage-gated Ca^2+^ channels and maintains homeostasis with other ions (Berridge et al. 1998). However, cellular membranes in AD can be altered by Aβ, which causes increasing Ca^2+^ influx and Ca^2+^-mediated excitotoxicity (Bezprozvanny and Mattson 2008). Studies have reported that interneurons with calcium-binding proteins such as calretinin might overcome the excitotoxicity induced by increasing intracellular Ca^2+^ concentration (Mikkonen et al. 1999), whereas interneurons without calcium-binding proteins but expressing neurotransmitters like CCK and SST are more likely to degenerate in AD (Saiz-Sanchez et al. 2015).

### The Triggers for Alteration in Intrinsic and Synaptic Homeostasis During the Pathogenesis of AD

A question that remains to be addressed is why there is a persistent intrinsic hyperexcitability of specific populations of neurons such as CCK and SST cells in early AD. This could be due to specific mutations in intrinsic voltage-gated ion channels resulting in the observed hyperexcitability, for example, background “leak” potassium channels; however, we suggest that there are multiple factors involved, including alterations in the pre- and postsynaptic synaptic release machinery and the upregulated activity of P2Y1Rs on CR cells and astrocytes leading to disrupted network behavior.

Evidence from various in vitro and in vivo studies has shown that the production and secretion of Aβ into the extracellular space are regulated by presynaptic neuronal factors such as activity-dependent presynaptic firing rates, which has been hypothesized to enhance the neurotransmitter release as a result of prolonged synaptic vesicle docking to the presynaptic membrane caused by Aβ interaction with various synaptic proteins (Russell et al. 2012; Marsh and Alifragis 2018). Furthermore, endosomal proteolytic cleavage of *APP* and Aβ release at synaptic terminals is thought to affect neurotransmitter recycling via interference with clathrin-dependent endocytosis (Kamenetz et al. 2003; Cirrito et al. 2005; Marsh and Alifragis 2018). Blocking such neuronal activity has been shown to oppose the Aβ toxicity effect (Kamenetz et al. 2003; Palop and Mucke 2010). According to this hypothesis, these mechanisms enhance neurotransmitter release. In particular, evidence for this comes from studies focusing on glutamatergic synapses, where the enhanced extracellular Aβ was shown to increase spontaneous excitatory postsynaptic events and facilitate presynaptic glutamatergic release in neurons with low activity but not in neurons with high activity (Abramov et al. 2009). This pathologically elevated Aβ has been shown to prevent glutamate reuptake at synapses, resulting in increased levels of glutamate in the synaptic cleft (Li et al. 2009). This would have various impacts leading to postsynaptic glutamate receptor desensitization as well as affecting neighboring synapses and the neuronal support systems such as astroglia.

### Physiological Consequences of Alteration in Synaptic Excitatory–Inhibitory Homeostasis

Figure 9 illustrates our findings on and the proposed outcome of CA1 interneurons in late AD. We suggest that hyperexcited presynaptic glutamatergic networks from other cortical regions such as the entorhinal cortex, shown to be in an excitatory overdrive (Petrache et al. 2019), activate CA1 interneurons rendering them hyperexcited. With this assumption, an enhanced firing of CCK and SST cells in our model will ultimately enhance the release of neurotransmitter GABA, and since CCK and SST cells are specialized to fine-tune and provide dendritic inhibition to CA1 pyramidal cells, perhaps this overdrive of inhibitory function in early stages of AD is a protective mechanism, preventing CA1 pyramidal cell hyperexcitability that is shown to develop at midstages of the disease (Petrache et al. 2019), developing at ~ 6 months and correlating with a decline in CCK and SST cell function probably due to exocytotic death.

The changes in these networks perhaps contributes as a “compensatory” mechanism to enhance CR interneuron function that we observe, since these cells in the rodent are located in all layers of the hippocampus. Two populations of CR cells have been suggested to exist in CA1: CR cells that contact CCK interneurons and CR cells colocalized with a neuropeptide, vasoactive intestinal peptide (VIP), which prefer to make synaptic contact with SST-expressing cells (Katona et al. 1999) but, overall, form synapses exclusively with dendrites of other interneurons, such as CCK and SST cells (Cauli et al. 2014, Tyan et al. 2014, Chamberland and Topolnik 2012), as well as electrically with each other via gap junctions (Gulyas et al. 1996). Thus, any alterations in the CCK and SST networks in later stages of AD will severely impact CA1 disinhibition, since electrical coupling mediated by gap junctions is thought to play a role in the generation of highly synchronized electrical activity (Traub et al. 2001a, Traub et al. 2001b). However, we suggest that the enhanced function of CR cells is *more* than a compensatory mechanism, but which is due to neuron–astrocyte interaction via enhanced P2Y1R activity, particularly in the later stages of AD. Interestingly, we observed an unexpected voltage-dependent increase in sIPSP frequency in the AD model, which could be explained by the altered presynaptic release machinery during the pathogenesis of AD, the presence of extracellular Aβ accumulation, and/or the involvement of a third party such as the proliferated reactive astrocytes.

It has been well documented that astrocyte hyperactivity is prominent around Aβ plaques and produces synchronous hyperactivity in [Ca^2+]^i transients across long distances that is uncoupled from neuronal activity (Kuchibhotla et al. 2009; Delekate et al. 2014). The nucleotides ATP and ADP released during proinflammatory responses potentiate the activity of reactive astrocytes in the APPPS1 AD mouse model, which has been suggested to be predominantly mediated by P2Y1Rs, which when activated cause an enhancement in spontaneous astrocyte calcium events (Delekate et al. 2014; Reichenbach et al. 2018). Blockade of these metabotropic P2Y1Rs on astrocytes after chronic treatment with an antagonist of P2Y1Rs normalizes the aberrant hyperactivity of reactive astrocytes and reduces neuronal hyperactivity and improves performance in the spatial memory test (Reichenbach et al. 2018). Others have also evidenced that reactive astrocytes either aberrantly produce the inhibitory gliotransmitter GABA (Jo et al. 2014) or release ATP regulating the excitability of CCK interneurons through P2Y1 receptor activation (Tan et al. 2017). It is conceivable that both of these mechanisms could also increase CR cell hyperinhibition in our system, which is yet to be determined.

Therefore, it is conceivable that the enhanced network activity of reactive astrocytes that proliferate in the later stages of AD is a result of “spillover” of glutamate from neighboring excitatory cells leading to an intracellular rise in calcium levels in astroglia leading to further release of glutamate promoting CR cell excitability, which was demonstrated previously in pyramidal neurons (Fellin et al. 2004, Jourdain et al. 2007).

Perhaps this cascade of events in addition to the upregulated P2Y1Rs results in enhanced activity of CR cells, which was normalized by blocking P2Y1Rs. In addition, the aberrant hyperexcitability of principal pyramidal cells was differentially affected, restoring the aberrant hyperexcitability of pyramidal cells and restoring the inhibition observed in control wild-type mice.

Interestingly, others have shown in healthy rodent brain that the activation of P2Y1Rs did not change the membrane effects in principal cells, in contrast to what we report here. Perhaps this difference could be due to the pathogenesis of AD. Furthermore, previous studies also report that the activation of P2Y1Rs on some interneurons causes a nonselective cationic current through the activation of transient receptor potential channels and the suppression of a resulting K^+^ conductance, resulting in membrane depolarization of these interneurons (by ~ 10 mV). As a result, the interneurons are thought to increase their firing frequency giving rise to an increased inhibition onto principal cells (Bowser and Khakh 2004; Kawamura et al. 2004). Therefore, we suggest that blocking of P2Y1Rs on CR cells “normalizes” the aberrant excitatory–inhibitory imbalance in AD through the blockade of CR network–associated excess of disinhibition. As a result, this allows other interneurons that were “supressed” by the CR network, such as CCK cells, to perform their intended function of directly inhibiting principal cells (Ali 2007), thus relieving the overexcitation of principal cells.

In summary, our data provides further evidence that AD pathogenesis involves complex synaptic mechanisms that lead to neurodegeneration rather than simple synaptic loss. This study demonstrates that Aβ affects excitatory and inhibitory synapses differentially but also the astrocytes and their receptors, which leads to complex synaptic imbalances in circuit and network activity. The paradoxical overexcitation observed over various cortical regions in a time-dependent fashion in AD (Petrache et al. 2019) may be related to changes in neuronal structures and their junctions of communication as suggested previously (Palop et al., 2006), but to date, the precise neuronal elements and their cellular mechanisms need to be further investigated. We show for the first time a cell-type–specific neuronal destruction and Aβ penetration and that interneuron-specific networks play an important role in altering the synaptic homeostasis of inhibition in CA1 through upregulated P2Y1Rs, and we suggest that hippocampal network dysfunction is more than a compensatory response, but due to underlying mechanistic interactions between Aβ and alterations of the expression of inhibitory neuropeptides and receptors, we propose that these data have important implications for future drug development of novel targeted therapy for AD.

## Funding

Wellcome Trust (United Kingdom); Medical Research Council (United Kingdom) New Investigators award (GO501263 to A.B.A); Medical Research Council PhD studentship (MR/N013867/1).

## Notes

The authors would like to thank Dr Andrew Constanti (UCL School of Pharmacy, United Kingdom) for his invaluable comments in the preparation of this manuscript. We would like to thank Profs Takashi Saito and Takaomi C. Saido, RIKEN Center for Brain Science, Japan, for the *App^NL-F/NL-F^* mouse model of AD. *Conflict of Interest*: None declared.

## Author Contributions

Anqi Shi: Performed the neuroanatomical studies, confocal microscope imaging, and data analysis of cholecystokinin and somatostatin cells and contributed in preparing the article. Alexandra Petrache: Performed the neuroanatomical studies, confocal microscope imaging, and data analysis of calretinin cells and contributed in preparing the article. Jiachen Shi: Performed with the confocal analysis of P2Y1 receptor colocalization and contributed in preparing the article. Afia B. Ali: Designed and coordinated the project, performed all electrophysiological whole-cell recordings, performed and supervised neuroanatomical studies, performed the data analysis, and prepared the article.
